# Synthesis, Characterization, and Biological Evaluation of *N*-Methyl Derivatives of Norbelladine

**DOI:** 10.3390/molecules29184442

**Published:** 2024-09-19

**Authors:** S. Mahsa Hashemian, Natacha Merindol, Alexis Paquin, Amita Singh, Lionel Berthoux, Benoit Daoust, Isabel Desgagné-Penix

**Affiliations:** 1 Department of Chemistry, Biochemistry and Physics, Université du Québec à Trois-Rivières, Trois-Rivières, QC G8Z 4M3, Canada; s.mahsa.hashemian@uqtr.ca (S.M.H.); natacha.merindol@uqtr.ca (N.M.); benoit.daoust@uqtr.ca (B.D.); 2 Department of Chemistry, Université du Québec à Montréal, Montréal, QC H2L 2C4, Canada; paquin.alexis@courrier.uqam.ca; 3 Department of Medical Biology, Université du Québec à Trois-Rivières, Trois-Rivières, QC G8Z 4M3, Canada; amita.singh@uqtr.ca (A.S.); lionel.berthoux@uqtr.ca (L.B.); 4 Plant Biology Research Group, Trois-Rivières, QC G8Z 4M3, Canada

**Keywords:** Amaryllidaceae alkaloids, tertiary amines, butyrylcholinesterase, acetylcholinesterase, RNA virus, molecular docking

## Abstract

Norbelladine derivatives have garnered attention in recent years due to their diverse biological activities and pivotal role in the biosynthetic pathway of Amaryllidaceae alkaloids. This study reports the synthesis and biological evaluation of four O,*N*-methylated derivatives of norbelladine. These derivatives were synthesized through a three-step process: forming imine intermediates from benzaldehydes with tyramine, hydrogenating them to secondary amines, and *N*-methylating these amines. The products were purified and characterized by ^1^H and ^13^C NMR spectroscopy. Their biological activities were assessed by evaluating their ability to inhibit Alzheimer’s disease-related enzymes acetylcholinesterase and butyrylcholinesterase. Additionally, the cytotoxic activity of the novel derivatives was tested against cancer cell lines derived from hepatocarcinoma (Huh7), adenocarcinoma (HCT-8), and acute myeloid leukemia (THP-1) cells, and their antiviral properties against a human coronavirus (HCoV-OC43), a flavivirus (dengue virus), and a lentivirus (pseudotyped HIV-1). Docking analysis was performed to understand the impact of the *N*-methylation on their pharmacological relevance. The results indicate that while *N*-methylation does not significantly affect antiviral activity, it enhances butyrylcholinesterase inhibition for *N*-methylnorbelladine and 4′-*O*,*N*-dimethylnorbelladine. Overall, this work enhances our understanding of norbelladine derivatives, provides new tools for Alzheimer’s disease research, and lays the groundwork for future pharmaceutical developments.

## 1. Introduction

Neurodegenerative disorders, cancer, and viral diseases increasingly affect the aging and growing human population, creating an urgent need for new molecules to combat these conditions. Plants produce specialized metabolites to adapt to unique conditions and defend against biotic and abiotic stresses. Interestingly, many of these metabolites also display therapeutic properties that benefit humans, explaining the long-standing use of medicinal plants to treat various diseases. Amaryllidaceae are a family of ornamental, medicinal, and edible plants, whose most common representatives include *Allium* and *Narcissus* species. Alkaloids isolated from the subfamily Amaryllidoideae are extensively studied for their biological properties. Amaryllidoideae alkaloids (AAs) exhibit a wide range of activities, including anticancer, antiviral, and anticholinesterase, making them valuable starting points for pharmacological research and drug development [1,2,3,4].

Norbelladine is a phenylethylamine-type alkaloid that serves as a key intermediate in the biosynthesis of more complex AAs [4,5]. The structure of norbelladine (C_15_H_17_NO_3_) consists of two aromatic rings connected by an ethylamine chain, which originates from the condensation of 3,4-dihydroxybenzaldehyde and tyramine [6]. This versatile and flexible scaffold allows for numerous modifications, such as methylation, oxidation, and cyclization. Downstream of the pathway, intramolecular oxidative coupling of 4′-*O*-methylnorbelladine leads to the formation of structurally diverse and biologically active AAs [4]. The most studied AA is probably galanthamine, which is used as a drug for the treatment of mild symptoms of neurodegenerative disorders such as Alzheimer’s disease (AD) [7]. The enzymes acetylcholinesterase (AChE) and butyrylcholinesterase (BuChE) play crucial roles in the pathophysiology of AD due to their involvement in the cholinergic system [8]. Galanthamine, an *N*-methylated AA, targets AChE, which is directly involved in the breakdown of acetylcholine, and its inhibition is a primary treatment strategy to alleviate symptoms. BuChE becomes increasingly relevant as the disease progresses, and its inhibition may offer additional therapeutic benefits [9]. 

Other AAs such as haemanthamine and lycorine were shown to exhibit potent anticancer activity [10,11]. In addition, effective antiviral AAs are continuously uncovered, particularly specific to RNA viruses of the *Flaviviridae* (e.g., Zika and dengue viruses) and *Coronaviridae* families [12,13,14,15,16,17,18]. 

**Figure 1:**
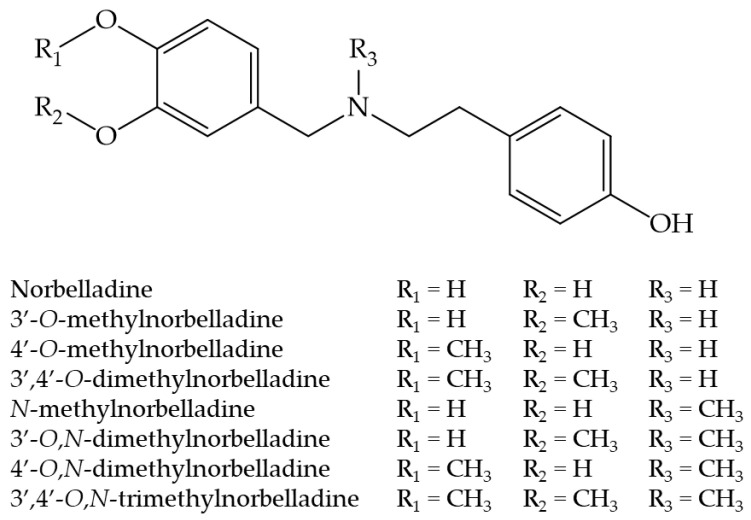
The chemical structure of norbelladine and its *O*- and *N*-methylated derivatives synthesized and investigated in this study.

Although isoquinoline and belladine-type alkaloids have been the subject of biological studies [19,20,21], research on the biological activity of norbelladine-type alkaloids remains limited. In a previous report, we uncovered that norbelladine and some *O*-methylated derivatives displayed anti-hepatocarcinoma activity, anti-BuChE, and anti-dengue virus activity [18]. To advance the characterization of norbelladine as a scaffold for drug development and to increase the biological properties of norbelladine derivatives, we methylated the ethylamine chain of these analogs and studied their biological properties. We investigated their anti-BuChE and anti-AChE properties as well as their toxicity and antiviral properties. Since plants usually do not store intermediate substances in an extractable amount, we produced these compounds through chemical synthesis. Figure 1 illustrates the chemical structure of the novel *N*-methylated derivatives of norbelladine that were synthesized and tested in this study. 

## 2. Results

### 2.1. Cytotoxic Activity

**Figure 2:**
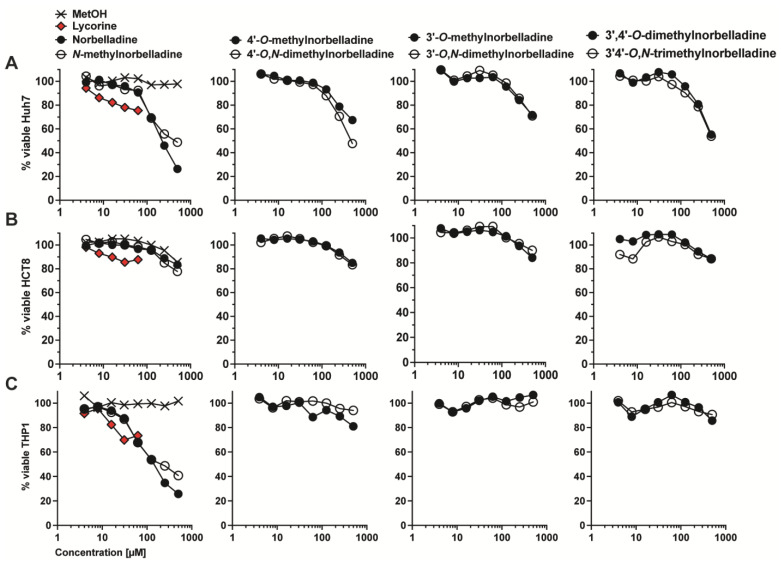
Cytotoxic properties of norbelladine *N*-methylated derivatives. Compounds were tested at concentrations ranging from 4–500 µM on Huh7 (**A**), HCT-8 (**B**), and THP-1 (**C**) cell lines. Methanol (MetOH) was used as solvent control, and lycorine as positive control. Levels were normalized to equivalent concentrations of MetOH. The *x*-axis is displayed in log_10_. The experiments were performed at least three times.

The cytotoxicity of norbelladine derivatives was evaluated against cancer cell lines derived from hepatocarcinoma (Huh7), adenocarcinoma (HCT-8), and acute myeloid leukemia (THP-1) following a 72 h treatment (Figure 2). HCT-8 cells showed low sensitivity to the compounds at concentrations ranging between 4 and 500 µM (Figure 2B). Norbelladine displayed modest cytotoxic activity toward THP-1 and Huh7 cells (Figure 2A,C). The *N*-methylation of norbelladine resulted in a slight decrease in the CC_50_ values for both cell lines (from 233 to 386 in Huh7, and from 148 to 227 µM in THP-1; Table 1). The *O* and *O*,*N*-di- and trimethylated derivatives were largely non-cytotoxic since the CC_50_ was not reached at 500 µM, except for 4′-*O*,*N*-dimethylnorbelladine (CC_50_ = 460.5 µM against Huh7 cells).

Overall, these results suggest that norbelladine derivatives are generally poorly cytotoxic to these cell lines, with norbelladine itself showing some anticancer activity, and that *N*-methylation does not significantly enhance this activity.

### 2.2. Antiviral Activity

**Figure 3:**
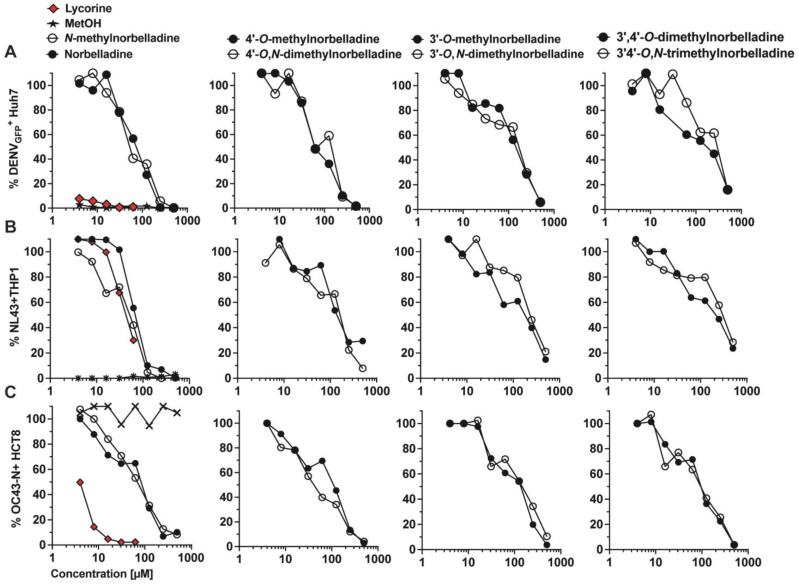
Antiviral properties of norbelladine *N*-methylated derivatives. Compounds were tested at concentrations ranging from 4–500 µM on dengue virus (DENV) (**A**), human immunodeficiency virus -1 (NL43) (**B**), and human coronavirus OC43 (OC43) (**C**) replication in Huh7, THP-1, and HCT-8 cell lines, respectively. Methanol (MetOH) was used as solvent control, and lycorine as positive antiviral control. Levels were normalized to infection levels in equivalent concentrations of MetOH. The *x*-axis is displayed in log_10_. The experiments were performed at least three times.

Previous studies have shown that norbelladine and its *O*-methylated derivatives possess detectable antiviral activity [18]. In this study, we evaluated the impact of *N*-methylation on the antiviral activity against flavivirus, lentivirus, and coronavirus (Figure 3, Table 1). Dengue virus reporter vector (DENV), pseudotyped human immunodeficiency virus (HIV-1), and wild-type human betacoronavirus OC43 (OC43) were used as representative models for their respective virus families. 

We confirmed that norbelladine-type alkaloids exhibit activity against DENV (Figure 3A) and HIV-1 infection (Figure 3B). Norbelladine, *N*-methylnorbelladine, 4′-*O*-methylnorbelladine, and 4′-*O*,*N*-dimethylnorbelladine were the most potent molecules against DENV replication with EC_50_ values ranging from 67.74 to 95.76 µM and selectivity index (SI) between 3.2 and >6.28 (Table 1). *N*-methylnorbelladine was more selective than norbelladine due to decreased cytotoxicity (SI of 5.7 vs. 3.3, respectively). Other derivatives had EC_50_ values exceeding 100 µM. *N*-methylation led to an increase in EC_50_ for 4′-*O*-methylnorbelladine and 3′,4′-*O*-dimethylnorbelladine, while it had minimal effect on other molecules.

For HIV-1, norbelladine and *N*-methylnorbelladine were the most active compounds, with EC_50_ values ranging between 66.89 and 42.28 µM, respectively. *N*-methylation led to an increased selectivity (from 2.22 to 5.36) (Figure 3B, Table 1). In the case of 3′-*O*-methylated derivatives, *N*-methylation resulted in an increased EC_50_ (Table 1).

We also uncovered that norbelladine derivatives inhibited betacoronavirus replication (Figure 3C, Table 1). The most potent compounds were 4′-*O*,*N*-dimethylnorbelladine, followed by norbelladine, 4′-*O*-dimethylnorbelladine, and *N*-methylnorbelladine, with EC_50_ values ranging between 42.37 and 66.77 µM, and SI values between >11.81 and >7.49. *N*-methylation of derivatives had no consistent impact on anticoronaviral activity.

### 2.3. Cholinesterase Inhibitory Effect

**Figure 4:**
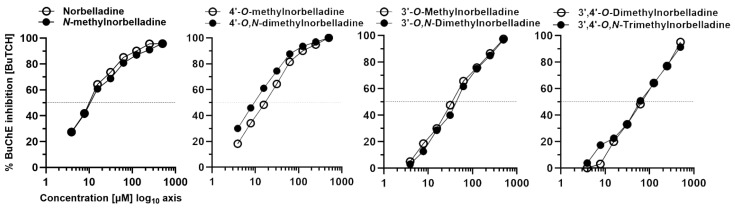
Butyrylcholinesterase (BuChE) inhibition by norbelladine and *N*-methylated derivatives using butyrylthiocholine (BuTCh) as the substrate.

Enzymatic inhibition experiments were carried out with *N*-methylated and non-*N*-methylated compounds as controls. Among the compounds tested (Figure 4, Table 2), *N*-methylnorbelladine and 4′-*O*,*N*-dimethylnorbelladine showed BuChE inhibition activity with IC_50_ values of 4 and 10.4 µM, respectively. The results indicate that *N*-methylation caused a 2-fold increase in BuChE inhibition for *N*-methylnorbelladine and 1.5-fold increase for 4′-*O*,*N*-dimethylnorbelladine compared to their respective non-*N*-methylated control (8 and 16.1 μM). Conversely, *N*-methylation increased the IC_50_ for 3′-*O*,*N*-dimethylnorbelladine and 3′,4′-*O*,*N*-trimethylnorbelladine compared to their non-*N*-methylated counterparts. 

Regarding AChE, none of the tested compounds inhibited this enzyme’s activity by more than 43.6% using acetylthiocholine as the substrate (Table 2). It is noteworthy that in the case of AChE, *N*-methylation created a similar trend to that observed for BuChE: *N*-methylation increased inhibition for *N*-methylnorbelladine and 4′-*O*,*N*-dimethylnorbelladine, while it decreased the inhibition for 3′-*O*,*N*-dimethylnorbelladine and 3′,4′-*O*,*N*-trimethylnorbelladine compared to non-*N*-methylated forms.

### 2.4. Molecular Docking of BuChE with Norbelladine Derivatives

**Figure 5:**
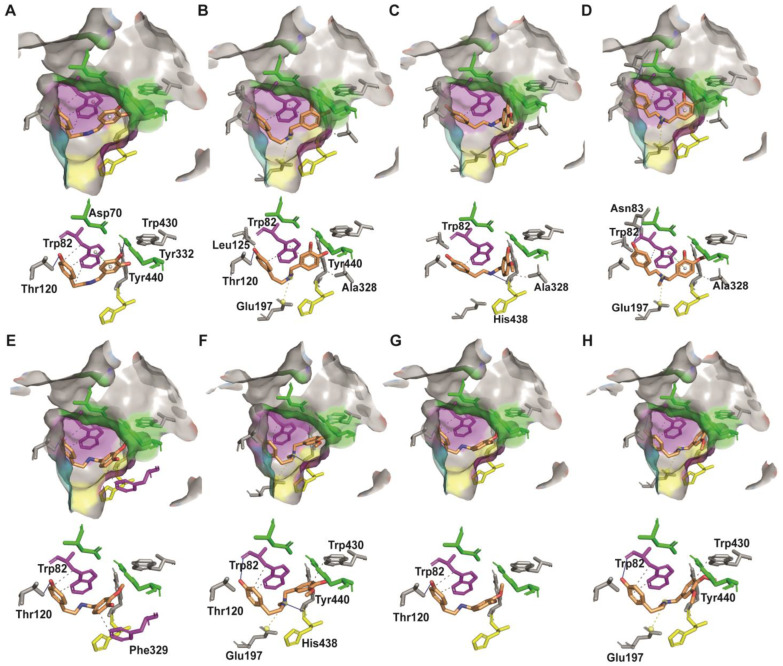
Docking of norbelladine derivatives with anti-butyrylcholinesterase activity. Grey surface representation of BuChE (4BDS) active site with catalytic triad in yellow, peripheral anionic site (PAS) in green, anionic site in pink, oxyanion pocket in turquoise, and the acyl pocket in dark grey. (**A**) Docked norbelladine in the active site pocket of BuChE and interacting residues. (**B**) Docked *N*-methylnorbelladine in the active site pocket of BuChE. (**C**). Docked 4′-*O*-methylnorbelladine in the active site pocket of BuChE. (**D**) Docked 4′-*O*,*N*-dimethylnorbelladine in the active site pocket of BuChE. (**E**) Docked 3′-*O*-methylnorbelladine in the active site pocket of BuChE. (**F**). Docked 3′-*O*,*N*-dimethylnorbelladine in the active site pocket of BuChE. (**G**). Docked 3′,4′-*O*-dimethylnorbelladine in the active site pocket of BuChE. (**H**) Docked 3′,4′-*O*,*N*-trimethylnorbelladine in the active site pocket of BuChE. H-bonds are shown as full blue lines, hydrophobic interactions as grey dashed lines, π-stacking interaction as green dashed lines in between white spheres, and salt bridges as yellow dashed lines in between yellow spheres.

To elucidate the role of *N*-methylation in the interaction between ligand and BuChE, molecular docking was performed using the crystal structure of human BuChE (PDB: 4BDS [22]) (Table 3, Figure 5). The docking scores were very similar for norbelladine and its derivatives, ranging from −6.42 to −7.4 kcal/mol (Table 3). All the molecules interacted with the key residue Trp82 at the anionic site. Norbelladine shared five bonds with this residue, and its interaction with BuChE was further supported by hydrogen bonds with the peripheral anionic site (PAS) amino acid Tyr332 and another binding site residue, Thr120. *N*-methylnorbelladine, 4′-*O*-methylnorbelladine, and 4′-*O*,*N*-dimethylnorbelladine interacted with another commonly reported key residue, Ala328, while others did not. 4′-*O*-methylnorbelladine and 4′-*O*,*N*-dimethylnorbelladine positioning was further supported by strong H-bond interaction with His438 and Asn83, respectively. A salt bridge interaction with Glu197 was observed for all *N*-methylated ligands. Interestingly, the distance between the N atom of *N*-methylnorbelladine and 4′-*O*,*N*-dimethylnorbelladine was smaller compared to 3′-*O*,*N*-dimethylated derivatives that showed decreased activity.

## 3. Discussion

Several subtypes of Amaryllidaceae alkaloids display diverse biological activities. Norbelladine and its derivatives are comparatively poorly investigated due to several factors such as limited natural occurrence, complex extraction from natural sources, and historical focus on other alkaloids. Previous initial studies have uncovered that some compounds of this scaffold displayed anti-inflammatory [23], antiviral [18], and anticholinesterase [24] activities. However, the impact of *N*-methylation on norbelladine and its *O*-methylated derivatives was previously unknown. 

Our study confirms that these compounds possess a slight but significant antiviral activity against representatives of three families of viruses, namely *Flaviviridae*, *Coronaviridae* and *Retroviridae.* Norbelladine, 4′-*O*-methylnorbelladine and their *N*-methylated form were the most active compounds. *N*-methylation did not significantly affect their antiviral potential. In most cases, 3′-*O*-methylation of norbelladine or 4′-*O*-methylnorbelladine in combination with their *N*-methylation resulted in decreased antiviral activity. This suggests that adding methyl groups at specific positions may alter interactions with the target. Further studies are needed to identify the targets and understand the key interactions required for inhibition. At this stage, the protein(s) that is(/are) targeted by norbelladine derivatives, viral or cellular, is unknown. Additionally, these compounds were generally non-cytotoxic, preventing a precise estimation of the SI and suggesting they do not possess anti-hepatocarcinoma, anti-myeloid, or anti-adenocarcinoma properties. The molecular targets of Amaryllidaceae alkaloids that cause their cytotoxicity have not been clearly identified, slowing down structure optimization for the development of anti-cancer treatment.

The investigation of the inhibitory effects of alkaloids on AChE and BuChE activity revealed that their potencies are significantly influenced by *O*- and *N*-methylation of their scaffold [19,24]. In our experiments, norbelladine and its *O*,*N*-methylated derivatives consistently inhibited the BuChE-catalyzed hydrolysis of butyrylcholine. *N*-methylnorbelladine was the most potent (IC_50_ = 3.94 μM), showing twice as much BuChE inhibition compared to norbelladine itself. 4′-*O*,*N*-dimethylnorbelladine showed 1.5-fold more BuChE inhibitory activity compared to its non-*N*-methylated counterpart. These findings are consistent with Mamun et al.’s [24] and Vaněčková et al.’s [19] study, underscoring the importance of the tertiary amine structure as a foundational element in designing butyrylcholinesterase inhibitors. Interestingly, the activity was specific to BuChE, as the *N*-methylated derivatives exhibited low AChE inhibition, with a maximum of 40% reached by 3′,4′-*O*,*N*-trimethylnorbelladine. These results are in agreement with Vaněčková et al.’s [19], who measured weak anti-AChE activity for 6-*O*-demethylbelladine (3′,4′-*O*,*N*-trimethylnorbelladine) and 4′-*O*-demethylbelladine.

To further characterize the mechanism of inhibition of the derivatives, we studied the simulated interaction with the enzyme following docking. BuChE is a serine hydrolase that plays a role in cholinergic signaling. The mature BuChE consists of 574 amino acids, and its active site is situated at the bottom of a deep gorge (20 Å) [25]. Studies based on crystallized structure and computational modeling analysis show conserved π-π interaction with Trp82 and H-bonding with His438 for the most potent inhibitors [26]. Hydrogen bonds with a bond distance of less than 3.1 Å are considered strong [27]. Therefore, all tested ligands exhibited strong interactions with the enzyme, supporting ligand–protein interactions. 

For all *N*-methylated ligands, the same interactions with Trp82 key residues was predicted by docking in addition to interactions with residues of the acyl pocket, which, according to Nachon et al., are crucial for compounds inhibitory effect [22]. In the case of *N*-methylated compounds, a new salt bridge interaction with Glu197 was observed, and an interrelationship is observed between the distance of the salt bridge bond and the amount of inhibition; the longer the bond, the less inhibition, which may explain the effect of salt bridge on inhibition. This interaction likely explains the increased inhibitory effect for *N*-methylnorbelladine and 4′-*O*,*N*-dimethylnorbelladine, as salt bridges enhance stability by maintaining specific conformations [28]. Additionally, these ligands share more hydrophobic interactions compared to non-*N*-methylated compounds, especially with Ala328, which has been reported as a common residue in interaction with different inhibitors in BuChE [26]. Thus, the docking results align with the inhibition mechanisms observed in other known inhibitors, such as tacrine, suggesting stronger and more stable inhibition potential for *N*-methylnorbelladine and 4′-*O*,*N*-dimethylnorbelladine compared to their secondary amine form.

## 4. Conclusions

In conclusion, we investigated the impact of *N*-methylation of various norbelladine derivatives and gained insight into the biological effect of such modification. Our results demonstrate that *N*-methylated norbelladine analogs are non-toxic toward the tested cell lines and display modest antiviral activity against OC-43, DENV, and HIV-1. In some cases, *N*-methylation increased the SI compared to the unmethylated analog. Furthermore, we have shown that for norbelladine and 4′-*O*-methylnorbelladine, *N*-methylation enhances BuChE and AChE inhibition. These findings suggest that *N*-methylation can be a beneficial modification for improving the biological activity of norbelladine derivatives. This could guide the optimization of AAs and similar compounds for potential therapeutic applications, such as in the treatment of Alzheimer’s disease. Future studies should also assess if these compounds display activities against other targets relevant to Alzheimer’s disease, similar to the alkaloid huperzine A, which modulates the accumulation of amyloid-β peptide, the mitochondria function, and the Wnt signaling pathway [29].

## 5. Materials and Methods

### 5.1. Chemical Synthesis and Purification of Norbelladine N-Methyl Derivatives

Tyramine (Thermo Scientific, Waltham, MA, USA 98+%), 3,4-Dihydroxybenzaldehyde (Sigma-Aldrich, Milwaukee, WI, USA 97%), vanillin (Sigma-Aldrich, Milwaukee, WI, USA 99%), isovanillin (Acros Organics, Geel, AN, Belgium 98%), and 3,4-Dimethoxybenzaldehyde (Sigma-Aldrich, Milwaukee, WI, USA 99%) were purchased commercially and used as received. Solvents were distilled and dried before starting the reaction using standard methods [30]. Nuclear magnetic resonance (^1^HNMR and ^13^CNMR) spectra were obtained on a Bruker 400 MHz NMR apparatus. Samples were dissolved in dimethyl sulfoxide (DMSO)-*d*_6_ (Sigma-Aldrich, Milwaukee, WI, USA 99.9%), with the residual solvent signal used as an internal standard (δ 2.49 ppm for ^1^H NMR and 39.95 ppm for ^13^C NMR). Chemical shifts (δ) are expressed in parts per million (ppm), and coupling constants (*J*) are expressed in hertz (Hz). Multiplicities are indicated by the following abbreviations: s for singlet, d for doublet, t for triplet, and m for multiplet.

Three-step synthesis of *N*-methylated derivatives of norbelladine:

*N*-methylnorbelladine, 3′-*O*,*N*-dimethylnorbelladine, 4′-*O*,*N*-dimethylnorbelladine, and 3′,4′-*O*,*N*-trimethylnorbelladine were obtained using organic synthesis following a three-step reaction sequence as described below. The products were characterized via proton (^1^H NMR) and carbon nuclear magnetic resonance (^13^CNMR) spectroscopy.

**Step 1: Synthesis of the imine intermediates.**

The synthesis of the imine intermediates, including norcraugsodine, 3′-*O*-methylnorcraugsodine, 4′-*O*-methylnorcraugsodine, and 3′,4′-*O*-dimethylnorcraugsodine, was carried out according to the method outlined by Girard et al., with small modifications. In summary, an equimolar amount of the corresponding benzaldehyde and tyramine powders was dissolved in dichloromethane (20 mL) in the presence of activated 4A molecular sieve. The solution was stirred overnight (12 h, room temp.) to form the imine intermediate. The mixture was filtered through a plug of silica gel with dichloromethane as the eluent. The filtrate was concentrated under reduced pressure, and the resulting imines were obtained in good yields. The products were sufficiently pure and were used in the next step without further purification. All compounds are known and spectral data are consistent with spectral information reported in the literature [18].

**Step 2: Preparation of the secondary amine intermediates.**

Hydrogenation of the imines to produce the amine intermediates (norbelladine, 3′-*O*-methylnorbelladine, 4′-*O*-methylnorbelladine, and 3′,4′-*O*-dimethylnorbelladine) was performed following the established protocol [18]. In summary, the corresponding imine was dissolved in a mixture of ethyl acetate/methanol (9:1, 10 mL), and 30 mol% palladium on carbon (Pd/C 10%) was added to the reaction flask under nitrogen. Hydrogen gas was then bubbled into the mixture at three intervals (t = 0, 30, and 60 min) throughout the reaction. The reaction mixture was stirred for 3 h, with the completion of the reaction confirmed via TLC. Subsequently, the mixture was filtered through a plug of silica gel using an ethyl acetate/methanol mixture (4:1) as the eluent. The solvent was then evaporated under reduced pressure to collect the amines for further processing. The products were sufficiently pure and were used in the next step without further purification. All compounds are known, and spectral data are consistent with spectral information reported in the literature [18].

**Step 3: Preparation of the final tertiary amines.**

The *N*-methylation step was performed according to the protocol suggested by Zippilli et al. [31], with modifications. An equivalent amount of the secondary amine and formaldehyde (solution 37–40% *w*/*v*) were dissolved in 5 mL of methanol, and the solution was stirred at room temperature for 4 h. Subsequently, NaBH_4_ (1 equivalent) was added at 0 °C, and the mixture was stirred for an additional 5 h at room temperature. The reaction mixture was then filtered over Celite^®^, and the solvent was evaporated under reduced pressure. The resulting crude product was purified via silica gel column chromatography using a solvent system of DCM/MeOH/NH_4_OH (14:1:0.1), resulting in the desired tertiary amine with yields ranging from 60% to 96%.

***N*-methylnorbelladine (4-(2-(4-hydroxyphenyl)(methyl)amino)methyl)benzene-1,2-diol**: Step 1 was performed with 537 mg of 3,4-dihydroxybenzaldehyde (3.88 mmol) and tyramine (533 mg, 3.88 mmol) and yielded 0.99 g of Norcraugsodine (99%). Step 2 was performed with 150 mg of Norcraugsodine (0.58 mmol) and yielded 130 mg of Norbelladine (86%), ^1^H-NMR (400 MHz, DMSO-*d*_6_): 6.96–6.94 (d, 2H, ArH), 6.69–6.68 (d, 2H, ArH), 6.65–6.61 (m, 2H, ArH), 6.52–6.50 (m, 2H, ArH), 3.52 (s, 2H, ArCH_2_N), 2.63–2.61 (m, 2H, NCH_2_CH_2_Ar), 2.59–2.58 (m, 2H, NCH_2_CH_2_Ar). ^13^C-NMR (100 MHz, DMSO-*d*_6_): 155.87,145.39, 144.40, 131.74, 130.76, 129.83, 119.31, 116.04, 115.60, 115.48, 53.01, 51.04, and 35.21. Step 3 was performed with 267 mg of norbelladine (1 mmol) and afforded 160.2 mg of *N*-methylnorbelladine as a brownish powder (60%). 1H-NMR (400 MHz, DMSO-*d*_6_): 6.97–6.94 (d, 2H, ArH), 6.65–6.62 (d, 2H, ArH), 6.25–6.20 (m, 2H, ArH), 6.19–6.14 (m, 2H, ArH), 3.16 (s, 2H, ArCH_2_N), 2.67–2.54 (m, 2H, NCH_2_CH_2_Ar), 2.46–2.32 (m, 2H, NCH_2_CH_2_Ar), 2.10 (s, 3H, -NCH_3_) ppm. ^13^C-NMR (100 MHz, DMSO-*d*_6_): 155.26, 146.83, 145.49, 130.58, 129.97, 129.32, 118.73, 116.17, 114.90, 62.42, 58,76, 45.08, 41.59, and 32.20.
**4′-*O*,*N*-dimethylnorbelladine (5-(((4-hydroxyphenethyl)(methyl)amino)methyl)-2-methoxyphenol)**: Step 1 was performed with 561 mg of iso-vanillin (3-hydroxy-4-methoxybenzaldehyde) (3.68 mmol) and tyramine (506 mg, 3.68 mmol), resulting in 0.99 g of 4′-*O*-methylnorcraugsodine (99%), ^1^H NMR (200 MHz, DMSO-*d*_6_) *d*: 6.97–6.63 (7H, m, CH-Ar), 3.72 (3H, s, OMe), 3.54 (2H, s, Ar-CH_2_-NH), and 2.67–2.55 (4H, m, NH-CH_2_CH_2_-Ar); ^13^C NMR (200 MHz, DMSO-*d*_6_) *d*: 155.84, 146.75, 146.68, 134.04, 130.94, 129.82, 118.92, 115.83, 115.46, 112.38, 56.10, 53.00, 51.20, and 35.46. Step 2 was performed with 266 mg (0.97 mmol) of 4′-*O*-methylnorcraugsodine and yielded 212 mg of 4′-*O*-methylnorbelladine (80%). Step 3 was performed with 273 mg (1 mmol) of 4′-*O*-methylnorbelladine and yielded 245.7 mg of 4′-*O*,*N*-dimethylnorbelladine as a white powder (90%). 1HNMR (400 MHz, DMSO-*d*_6_): 6.91–6.47 (m, 7H, ArH), 3.70 (s, 3H, -OCH3), 3.30 (s, 2H, ArCH_2_N-), 2.60–2.41 (m, 4H, -NCH_2_CH_2_Ar), 2.11 (s, 3H, -NCH_3_) ppm. ^13^C-NMR (100 MHz, DMSO-*d*_6_): 156.06, 147.55, 146.72, 131.53, 129.75, 129.26, 118.20, 116.19, 115.09, 111.70, 61.06, 58.96, 55.47, 41.63, 32.17 ppm.
**3′-*O*,*N*-dimethylnorbelladine (5-(((3-hydroxyphenethyl)(methyl)amino)methyl)-2-methoxyphenol)**: Step 1 was performed with 561 mg of vanillin (4-hydroxy-3-methoxybenzaldehyde) (3.68 mmol) and tyramine (506 mg, 3.68 mmol), resulting in 0.83 g of 3′-*O*-methylnorcraugsodine (83%). Step 2 was performed with 182.5 mg of 3′-*O*-methylnorcraugsodine (0.67 mmol) and yielded 170.0 mg of 3′-*O*-methylnorbelladine (92%), ^1^H NMR (200 MHz, DMSO-*d*_6_) *d*: 6.98–6.61 (7H, m, CH-Ar), 3.80 (2H, s, Ar-CH_2_-NH), 3.71 (3H, s, OMe), and 2.67–2.61 (4H, m, NHCH_2_CH_2_-Ar); ^13^C NMR (200 MHz, DMSO-*d*_6_) *d*: 155.96, 147.92, 147.13, 130.27, 129.85, 124.69, 121.12, 118.48, 115.54, 111.59, 56.04, 50.83, 50.57, and 34.85. Step 3 was performed with 273 mg of 3′-*O*-methylnorbelladine (1 mmol) and yielded 245.7 mg of 4′-*O*,*N*-dimethylnorbelladine as a white powder (90%). ^1^HNMR (400 MHz, DMSO-*d*_6_): 6.98–6.64 (m, 7H, ArH), 3.81 (s, 2H, ArCH_2_N-), 3.72 (s, 3H, -OCH_3_), 2.70–2.60 (m, 4H, -NCH_2_CH_2_Ar), 2.08 (s, 3H, -NCH_3_) ppm. ^13^C-NMR (100 MHz, DMSO-*d*_6_): 155.98, 147.92, 147.10, 130.22, 129.86, 124.57, 121.17, 118.50, 115.55, 111.62, 56.04, 50.73, 50.54, 49.06, and 34.78.
**3′,4′-*O*,*N*-trimethylnorbelladine (5-(((3,4-dihydroxyphenethyl)(methyl)amino)methyl)-2-methoxyphenol)**: Step 1 was performed with 582 mg of 3,4-dimethoxybenzaldehyde (3.50 mmol) and tyramine (481 mg, 3.50 mmol), resulting in 0.56 g of 3′,4′-*O*-dimethylnorcraugsodine (56%). Step 2 was performed with 202 mg of 3′,4’-*O*-dimethylnorcraugsodine (0.7 mmol), resulting in 170 mg of 3′,4′-*O*-dimethylnorbelladine (83%), ^1^H NMR (200 MHz, DMSO-*d*_6_) *d*: 6.98–6.64 (7H, m, CH-Ar), 3.71 (6H, s, OMe), 3.65 (2H, s, Ar-CH2-NH), and 2.67–2.01 (4H, m, NH-CH_2_CH_2_-Ar); ^13^C NMR (200 MHz, DMSO-*d*_6_) *d*: 155.9, 149.03, 148.03, 133.35, 130.72, 129.86, 120.44, 115.47, 112.23, 111.95, 55.95, 55.79, 52.87, 50.92, and 35.15. Step 3 was performed with 273 mg of 3′,4′-*O*-dimethylnorbelladine (1 mmol) to yield 262.1 mg of 3′,4′-*O*,*N*-trimethylnorbelladine as a yellow powder (96%). ^1^HNMR (400 MHz, DMSO-*d*_6_): 6.98–6.62 (m, 7H, ArH), 3.71 (s, 3H, -OCH_3_), 3.62 (s, 2H, ArCH_2_N-), 2.66–2.55 (m, 4H, -NCH_2_CH_2_Ar), 1.89 (s,3H, -NCH_3_) ppm. ^13^C-NMR (100 MHz, DMSO-*d*_6_): 155.28, 148.44, 147.33, 133.19, 130.33, 129.27, 119.69, 114.96, 111.54, 111.34, 55.36, 52.44, 50.51, 48.47, 34.80, and 21.19.

**Figure A1:**
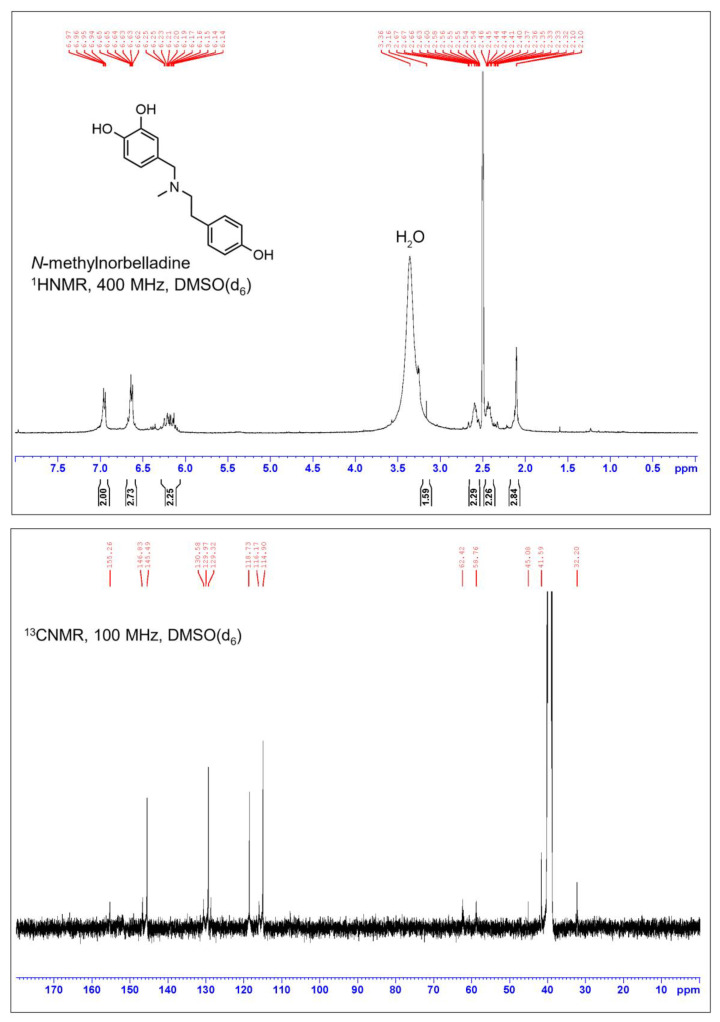
NMR spectra NMR spectra of *N*-methylnorbelladine.

**Figure A2:**
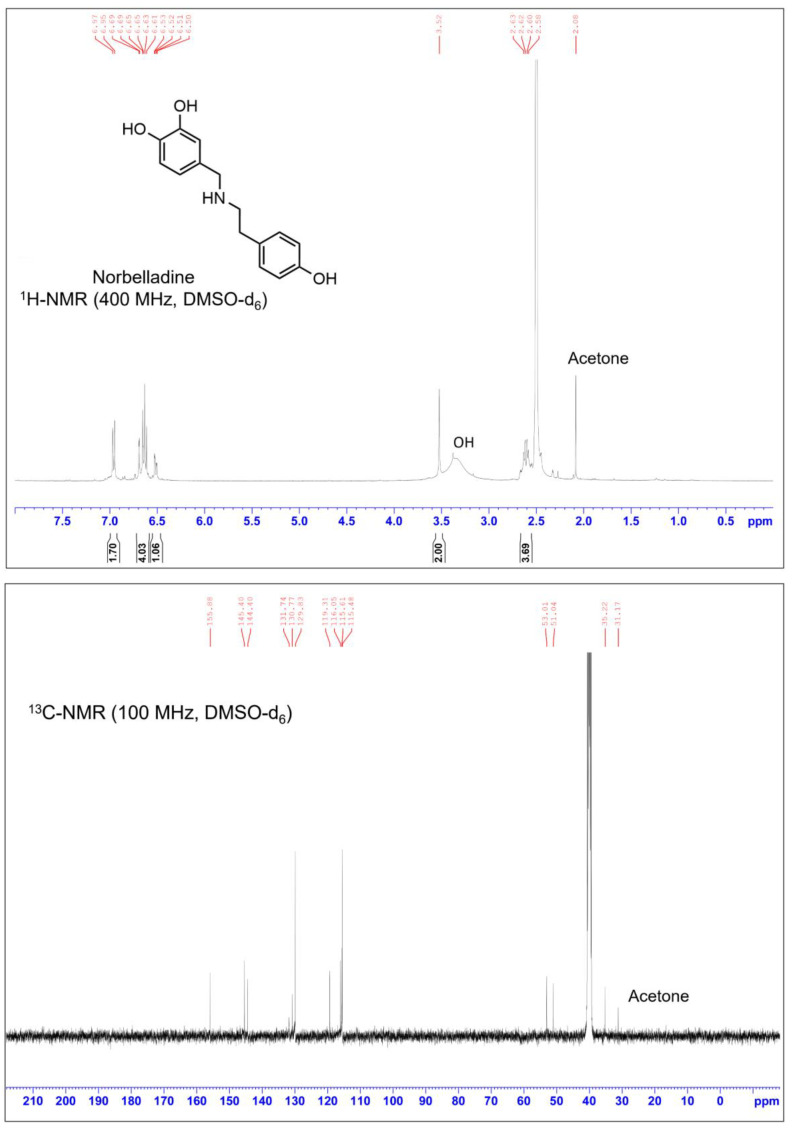
NMR spectra NMR spectra of norbelladine.

**Figure A3:**
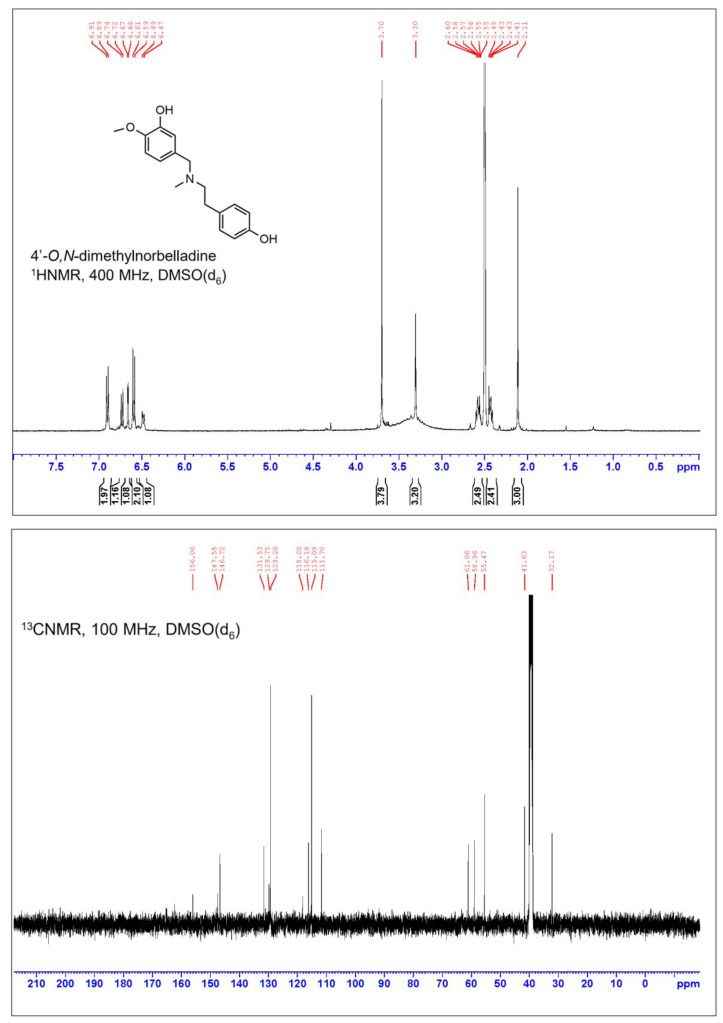
NMR spectra NMR spectra of 4′-*O*,*N*-dimethylnorbelladine.

**Figure A4:**
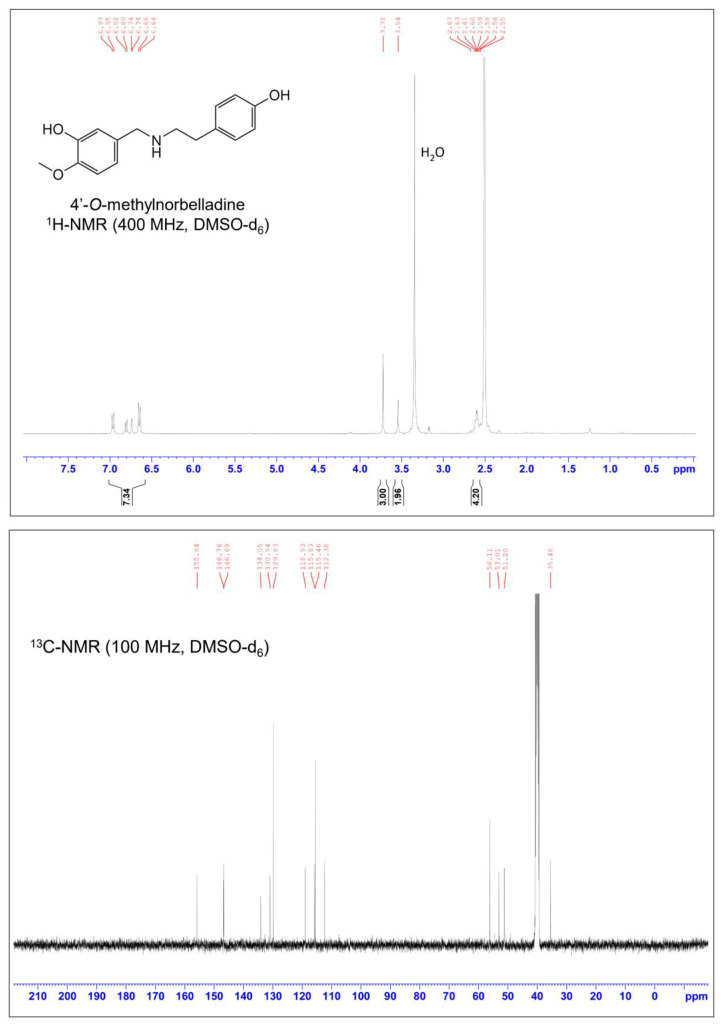
NMR spectra NMR spectra of 4′-*O*-methylnorbelladine.

The synthesized *N*-methylated norbelladine derivatives were purified and characterized by NMR spectroscopy (Appendix A, Figure A1, Figure A2, Figure A3 and Figure A4).

### 5.2. Anti-Acetylcholinesterase (AChE) and -Butyrylcholinesterase (BuChE) Activity

Pharmacological properties specific to Alzheimer’s disease (AD) were tested on acetylcholinesterases (AChEs) from electric eels and butyrylcholinesterase (BuChE) from equine species using a colorimetric kit (ab138871, Abcam, Cambridge, UK), which measures enzyme activity and inhibition. The reaction was carried out in a final volume of 100 µL in 96-well microplates. A preliminary screening identified the most potent AChE and BuChE inhibitors by adding test compounds dissolved in DMSO to a final concentration of 2 mM (1% DMSO) in duplicates. A 5 µL reaction mixture containing equal amounts of acetylthiocholine/butyrylthiocholine (20X) and DTNB (20X) was then added to each well. Subsequently, the enzyme solution was added to a final concentration of 2 U/mL, and absorbance was measured at 412 nm in kinetic mode for 10 min using a microplate reader (Synergy H1, Biotek, Dorval, QC, Canada). DTNB (Bis(3-carboxy-4-nitrophenyl) disulfide, Ellman’s Reagent), acetylthiocholine iodide, and butyrylthiocholine iodide were obtained from Sigma-Aldrich (Oakville, ON, Canada). Galanthamine (50–0.05 µM) and rivastigmine (400–0.4 µM) were used as positive controls for the AChE and BuChE assays, respectively. Compounds showing inhibition during preliminary screenings were selected for further IC_50_ value assessment using serially diluted concentrations. All experiments were performed at least twice. Inhibition was calculated using the formula [24]:

I = 100 × (1 − ΔAi/ΔA0),

where ΔAi is the difference in absorbance between two time points in the presence of the inhibitor, and ΔA0 is the difference in absorbance between two-time points in the presence of DMSO or an appropriate solvent.

### 5.3. Molecular Docking

Docking was performed according to Girard et al. [18]. To summarize, the crystal structure of human BuChE in a complex with tacrine (PDB: 4BDS) used to do the docking using MOE 2020.09 software (Chemical Computing Group). First, Tacrine was removed. The ligands and proteins were prepared and protonated at pH = 7 using the protomers tool. The active site (Asn68, Ile69, Asp70, Gln71, Ser72, Gly78, Ser79, Trp82, Tyr114, Gly115, Gly116, Gly117, Gln119, Thr120, Gly121, Thr122, Leu125, Tyr128, Glu197, Ser198, Ala199, Trp231, Glu276, Ala277, Val280, Gly283, Thr284, Pro285, Leu286, Ser287, Val288, Asn289, Phe290, Ala328, Phe329, Tyr332, Phe398, Trp430, Met437, His438, Gly439, Tyr440, Ile442) was predicted using the site finder tool of the MOE software and confirmed comparing to the literature [26]. Dummy atoms across the active site were created and used as docking sites. Water and solvent molecules were removed, residues further than 8 Å from dummy atoms were fixed, and active site residues were tethered using the QuickPrep default parameters. Induced fit was applied as a refinement option with 10 poses and the GBVI/WSA score. The best pose of the most abundant configuration was selected for protein–ligand interaction analysis. The Protein–Ligand Interaction Profiler (PLIP) was used to analyze ligand interactions with the binding site post-docking [32]. Visualization and presentation of the PLIP results were conducted using PyMOL (Schrödinger).

### 5.4. Cell Lines and Culture

The human hepatocarcinoma Huh7 cell line (CVCL_0336) was maintained in Dulbecco’s Modified Eagle Medium (DMEM) supplemented with 10% fetal bovine serum (FBS) and 1% penicillin/streptomycin solution (PS, from GIBCO, Fisher Scientific, Toronto, ON, Canada). The human leukemia monocytic THP-1 (CVCL_0006) and adenocarcinoma HCT-8 cell lines (CVCL_2478) were maintained in Roswell Park Memorial Institute (RPMI) medium supplemented with 10% FBS or horse serum, respectively, and 1% PS. All reagents and antibiotics were purchased from GIBCO, Fisher Scientific, Toronto, ON, Canada). Cells were kept in an incubator at 37 °C and 5% CO_2_.

### 5.5. Cytotoxicity Assay

In total, 10 × 10^3^ Huh7 cells/well, or 5 × 10^4^ THP-1 or HCT-8 cells/well were plated in 96-well plates and incubated at 37 °C and 5% CO_2_. Compounds (4–500 µM, 2-fold dilutions) were added the next day and cytotoxicity was assessed 72 h later by measuring ATP levels with the Cell-Titer GLO assay kit (Promega, Madison, WI, USA). Cell-Titer GLO reagent was added to the plates previously equilibrated to room temperature for 30 min. Plates were mixed for 2 min and incubated for 10 min at room temperature. The luminescence signal was measured with a microplate reader (Synergy H1, Biotek, Dorval, QC, Canada). Viability percentage was calculated as the ratio of the signal of cells exposed to compounds to the signal of cells exposed to solvent (methanol). All experiments were performed at least twice. Median cytotoxic concentrations (CC_50_) were calculated using GraphPad prism 10.0.2 (GraphPad Software, San Diego, CA, USA).

### 5.6. Viral Vectors

Wild type betacoronavirus HCoV-OC43 (Betacoronavirus 1, VR1558, ATCC), dengue virus propagative vector (DENV_GFP_), and a non-propagative human immunodeficiency virus (HIV)-1 pseudotyped VSV-G vector (HIV-1_GFP_) were included as representative of the betacoronavirus, the flavivirus, and the lentivirus geni. pFK-DVs-G2A encoding DENV_GFP_ vector was provided by Ralf Bartenschlager (Heidelberg University, Heidelberg, Germany) and Laurent Chatel-Chaix (Institut National de la Recherche Scientifique, Laval, QC, Canada) [15,33]. PMD2.G and pNL4-3-GFPΔEnvΔNef were used for the HIV-1_GFP_ vector [34]. DENV_GFP_ titer was measured using a plaque assay in Vero cells, as described in [35]. HIV-1_GFP_ titer was measured using serially diluted vectors on CRFK cells, as described in [15].

### 5.7. Antiviral Assays 

Cells were plated in 96 flat-bottom wells plate and treated with compounds exactly as for the cytotoxicity assay. Viral vectors (DENV_GFP_, HIV-1_GFP_, and HCoV-OC43 at multiplicity of infection (MOI) of 0.1 virus/ cell) were added 30–60 min following treatment and cells were incubated at 33 °C with 5% CO_2_ for 2 h on a rocking shaker prior to the 3 days incubation without rocking. Lycorine was used as positive control, methanol as solvent control. HIV-1_GFP_ infected THP-1 cells were resuspended and fixed in 4% formaldehyde. DENV_GFP_ infected Huh7 cells were washed twice with PBS, trypsinized, and resuspended in PBS with 4% formaldehyde. HCoV-OC43 infected HCT8 cells were washed twice, trypsinized, and washed twice in antibody dilution buffer (PBS + 0.5% BSA (Sigma-Aldrich, Milwaukee, WI, USA)) via centrifugation 180× *g* for 5 min. Cells were fixed in 4% formaldehyde for 15 min, washed twice in antibody dilution buffer and permeabilized in antibody dilution buffer containing 0.03% Triton X-100 for 10 min. Cells were washed twice and incubated with 1:1000 anti-HCoV-OC43 nucleoprotein antibody (1:1000; clone 542-7D; Sigma-Aldrich, Milwaukee, WI, USA) for 1 h at room temperature and overnight at 4C. Cells were washed once and incubated with 1:400 chicken anti-mouse CF488 secondary antibody (Millipore Sigma, Burlington, MA, USA) for 60 min at room temperature. Cells were washed and resuspended in antibody dilution buffer containing 1: 10,000 Hoechst 33362 (Thermo Fisher, Saint-Laurent, QC, Canada). The percentages of virus-infected cells were analyzed on a Cytoflex S flow cytometer (Beckman) equipped with 405 and 488 nm lasers, and Hoechst^+^ and GFP^+^ cells were analyzed in the 450/45 BP the 525/40 BP channels, respectively. Data analysis was performed using the Flowjo software (version 10.10, BD, FlowJo LLC, Ashland, OR, USA). All experiments were performed at least twice. 

### 5.8. Statistical Analysis

The graphs and analyses of the biological assays, including EC_50_ and CC_50_ values, were calculated using GraphPad Prism version 10.0.2 (GraphPad Software, San Diego, CA, USA).

## Figures and Tables

**Table 1:** Cytotoxic and antiviral properties of norbelladine and *O*- and *N*-methylated derivatives.

	CC_50_ (µM)			EC_50_ (µM)			SI		
	HCT-8	Huh7	THP-1	OC-43	DENV	HIV-1	OC-43	DENV	HIV-1
Norbelladine	>500	233.1	148.5	55.71	70.63	66.89	>8.98	3.30	2.22
*N*-methylnorbelladine	>500	386.2	226.6	66.77	67.74	42.28	>7.49	5.70	5.36
4′-*O*-methylnorbelladine	>500	>500	>500	63.04	79.56	163.2	>7.93	>6.28	>3.06
4′-*O*,*N*-dimethylnorbelladine	>500	460.5	>500	42.37	95.76	123.3	>11.80	4.81	>4.06
3′-*O*-methylnorbelladine	>500	>500	>500	74.55	138.3	176.7	>6.71	>3.62	>2.83
3′-*O*,*N*-dimethylnorbelladine	>500	>500	>500	118.7	126.5	244.2	>4.21	>3.95	>2.05
3′,4′-*O*-dimethylnorbelladine	>500	>500	>500	84.67	137.3	189.9	>5.91	>3.64	>2.63
3′,4′-*O*,*N*-trimethylnorbelladine	>500	>500	>500	81.96	296.7	287.2	>6.10	>1.69	>1.74

Abbreviations: CC_50_: concentration associated with 50% of cell death; EC_50_: concentration associated with 50% of viral inhibition; SI: selectivity index as the ratio of CC_50_/EC_50_; OC43: human coronavirus OC43; DENV: dengue virus. In several cases, the SI could not be determined due to lack of cytotoxicity at the tested concentrations.

**Table 2:** BuChE and AChE inhibition percentage at the highest tested concentration (500 μM) and IC_50_ calculated for all *N*-methylated compounds compared to non-*N*-methylated compounds as control.

Compound	% BuChE Inhibition (500 μM)	BuChE IC_50_ (μM)	% AChE Inhibition (500 μM)	AChE IC_50_ (μM)
Norbelladine	95	8	24.5	Nd
*N*-methylnorbelladine	96	4	31.2	Nd
4′-*O*-methylnorbelladine	99	16.1	33.62	Nd
4′-*O*,*N*-dimethylnorbelladine	99	10.4	43.6	Nd
3′-*O*-methylnorbelladine	96	27.5	10	Nd
3′-*O*,*N*-dimethylnorbelladine	96	38	0	Nd
3′,4′-*O*-dimethylnorbelladine	94	108.3	37	Nd
3′,4′-*O*,*N*-trimethylnorbelladine	91	127	25.5	Nd

Abbreviations: Nd not determined (>500 μM): because inhibition did not reach >50%; IC_50_ could not be determined.

**Table 3:** Predictions of norbelladine derivatives’ interactions with butyrylcholinesterase.

Ligand	Score (kCal/mol)	Interactions							
		Hydrophobic		H-Bond		π Stack		Salt Bridge	
		res	dist (Å)	res	dist (Å)	res	dist (Å)	res	dist (Å)
**Norbelladine**	−6.5	Trp82 (n = 4), Tyr440	3.48, 3.96, 3.98, 3.67 3.68	Thr120, Tyr332 (n = 2)	2.55, 2.30 2.42	Trp82	3.65	n.d.	n.a.
***N*** **-methylnorbelladine**	−6.4	Trp82 (n = 2), Leu125, Ala328, Tyr440	3.47, 3.63 4.00, 3.71 3.95	Thr120	2.59	n.d.	n.a.	Glu197	4.18
**4′-*O*-methylnorbelladine**	−6.6	Trp82, Ala328	3.36, 3.51	His438	3.44	n.d.	n.a.	n.d.	n.a.
**4′-*O*,*N*-dimethylnorbelladine**	−6.9	Trp82, Ala328	3.82, 3.98	Asn83	3.24	Trp82	4.86	Glu197	4.25
**3′-*O*-methylnorbelladine**	−7.0	Trp82 (n = 2), Phe329	3.55, 3.95 3.70	Thr120	2.62	n.d.	n.a.	n.d.	n.a.
**3′-*O*,*N*-dimethylnorbelladine**	−7.1	Trp82 (n = 2), Trp430, Tyr440	3.52, 3.82 3.67, 3.81	Trp82, His438	2.24, 3.11	n.d.	n.a.	Glu197	4.95
**3′,4′-*O*-dimethylnorbelladine**	−7.4	Trp82 (n = 2),	3.58, 3.96	Thr120	2.61	n.d.	n.a.	n.d.	n.a.
**3′,4′-*O*,*N*-trimethylnorbelladine**	−7.3	Trp82 (n = 2), Trp430, Tyr440	3.60, 3.71, 3.73, 3.57	Trp82	2.04	n.d.	n.a.	Glu197	4.66

Res: residue; dist: distance; n.d.: not detected; n.a.: not applicable.

**Figure A5:**
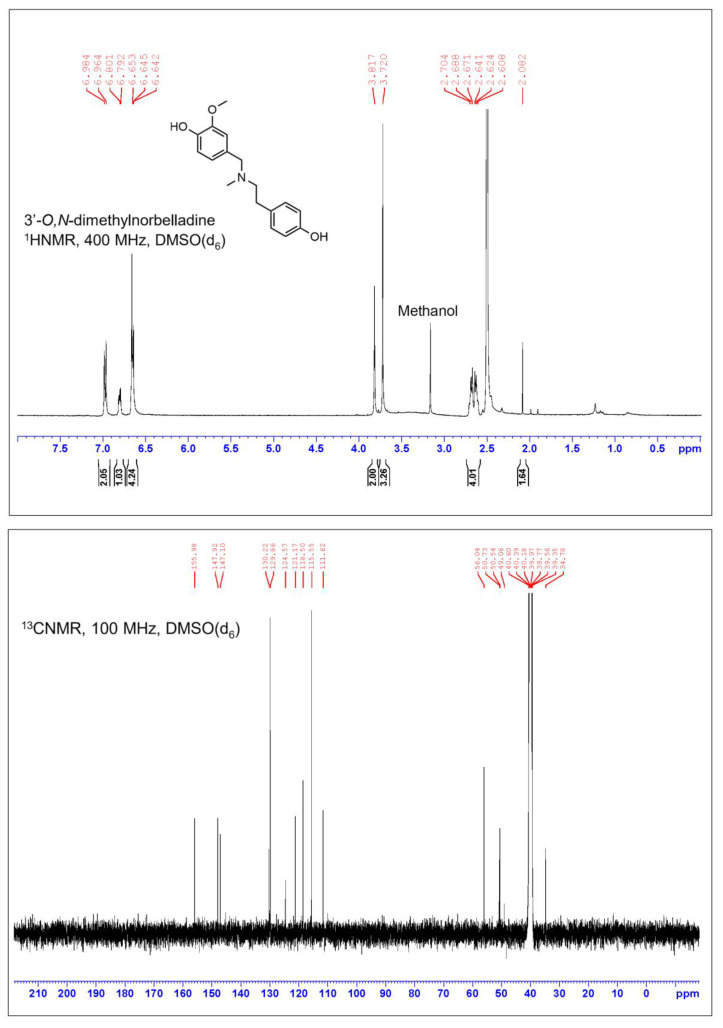
NMR spectra NMR spectra of 3′-*O*,*N*-dimethylnorbelladine.

**Figure A6:**
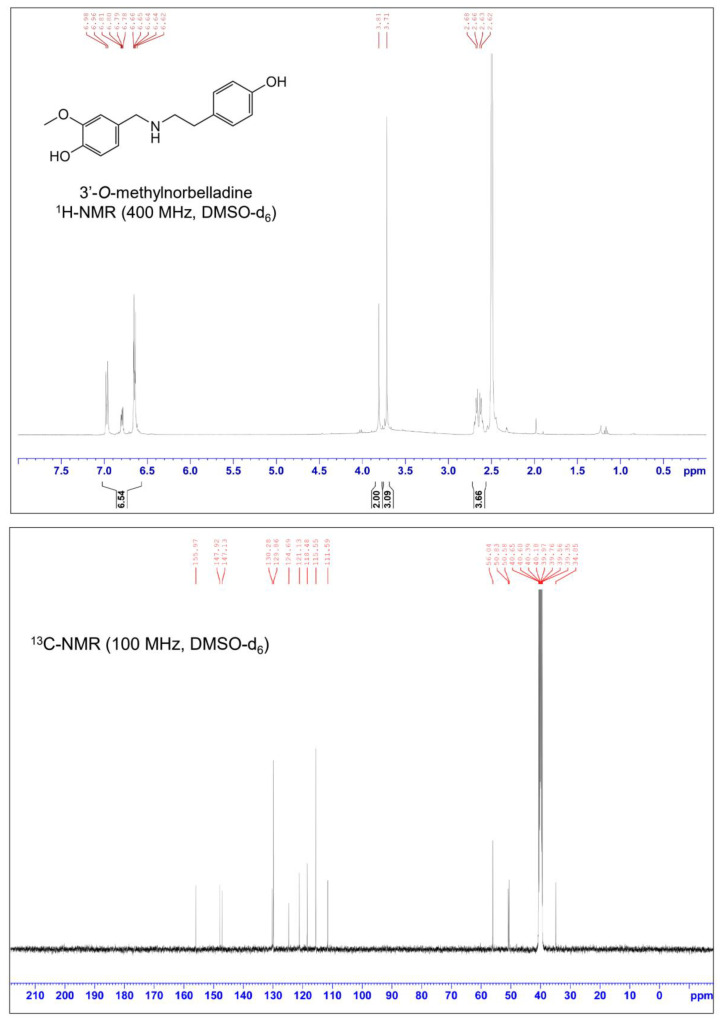
NMR spectra NMR spectra of 3′-*O*-methylnorbelladine.

**Figure A7:**
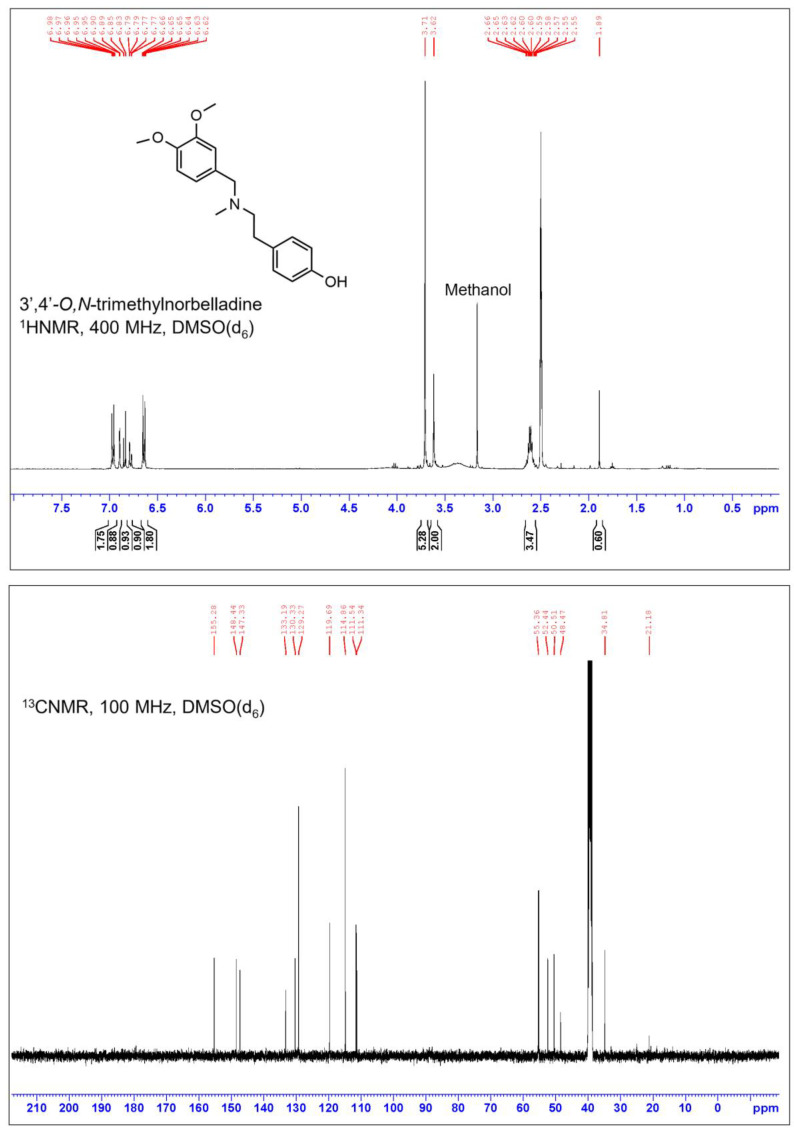
NMR spectra NMR spectra of 3′,4′-*O*,*N*-trimethylnorbelladine.

**Figure A8:**
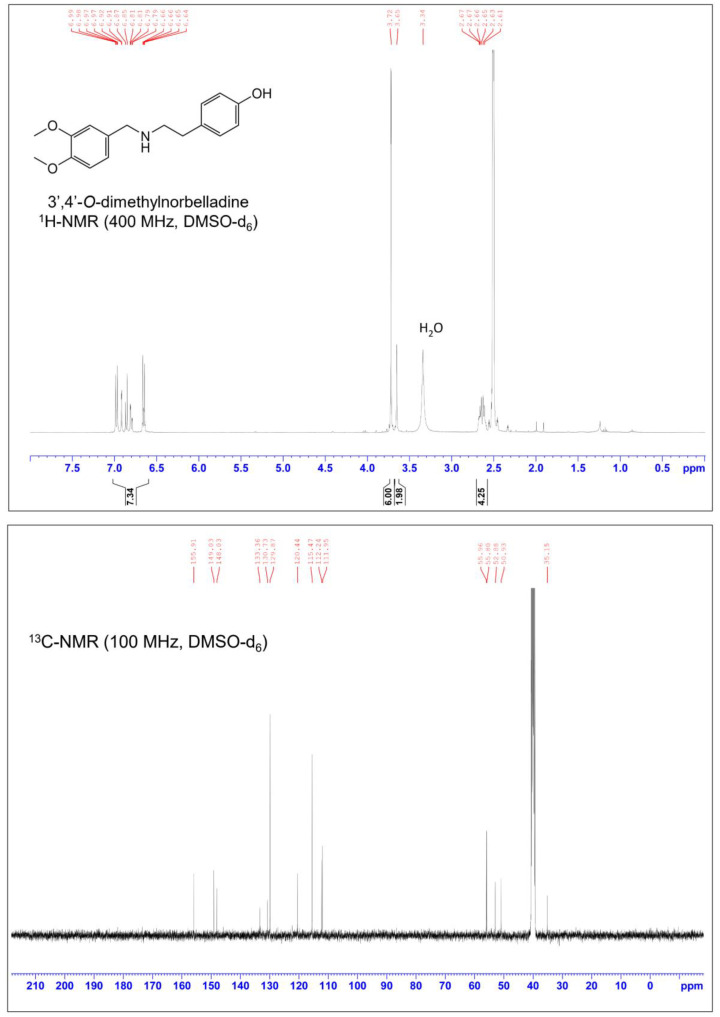
NMR spectra NMR spectra of 3′,4′-*O*-dimethylnorbelladine.

## Data Availability

The authors confirm that the data supporting the findings of this study are available within the article.

## References

[1] Jin Z., Xu X.-H., Ramawat K.G., Mérillon J.-M. (2013). Amaryllidaceae Alkaloids. Natural Products: Phytochemistry, Botany and Metabolism of Alkaloids, Phenolics and Terpenes.

[2] Hotchandani T., Desgagne-Penix I. (2017). Heterocyclic Amaryllidaceae Alkaloids: Biosynthesis and Pharmacological Applications. Curr. Top. Med. Chem..

[3] Desgagné-Penix I. (2021). Biosynthesis of alkaloids in Amaryllidaceae plants: A review. Phytochem. Rev..

[4] Jayawardena T.U., Merindol N., Liyanage N.S., Desgagné-Penix I. (2024). Unveiling Amaryllidaceae alkaloids: From biosynthesis to antiviral potential—A review. Nat. Prod. Rep..

[5] Singh A., Massicotte M.-A., Garand A., Tousignant L., Ouellette V., Bérubé G., Desgagné-Penix I. (2018). Cloning and characterization of norbelladine synthase catalyzing the first committed reaction in Amaryllidaceae alkaloid biosynthesis. BMC Plant Biol..

[6] Lichman B.R. (2021). The scaffold-forming steps of plant alkaloid biosynthesis. Nat. Prod. Rep..

[7] Heinrich M., Lee Teoh H. (2004). Galanthamine from snowdrop—The development of a modern drug against Alzheimer’s disease from local Caucasian knowledge. J. Ethnopharmacol..

[8] Bartolucci C., Perola E., Pilger C., Fels G., Lamba D. (2001). Three-dimensional structure of a complex of galanthamine (Nivalin) with acetylcholinesterase from Torpedo californica: Implications for the design of new anti-Alzheimer drugs. Proteins.

[9] Cummings J., Lee G., Zhong K., Fonseca J., Taghva K. (2021). Alzheimer’s disease drug development pipeline: 2021. Alzheimer’s Dement..

[10] Evidente A., Kireev A.S., Jenkins A.R., Romero A.E., Steelant W.F., Van Slambrouck S., Kornienko A. (2009). Biological evaluation of structurally diverse amaryllidaceae alkaloids and their synthetic derivatives: Discovery of novel leads for anticancer drug design. Planta Med..

[11] Pellegrino S., Meyer M., Zorbas C., Bouchta S.A., Saraf K., Pelly S.C., Yusupova G., Evidente A., Mathieu V., Kornienko A. (2018). The Amaryllidaceae Alkaloid Haemanthamine Binds the Eukaryotic Ribosome to Repress Cancer Cell Growth. Structure.

[12] Gabrielsen B., Monath T.P., Huggins J.W., Kefauver D.F., Pettit G.R., Groszek G., Hollingshead M., Kirsi J.J., Shannon W.M., Schubert E.M. (1992). Antiviral (RNA) activity of selected Amaryllidaceae isoquinoline constituents and synthesis of related substances. J. Nat. Prod..

[13] Wang P., Li L.F., Wang Q.Y., Shang L.Q., Shi P.Y., Yin Z. (2014). Anti-dengue-virus activity and structure-activity relationship studies of lycorine derivatives. ChemMedChem.

[14] Zhang Y.N., Zhang Q.Y., Li X.D., Xiong J., Xiao S.Q., Wang Z., Zhang Z.R., Deng C.L., Yang X.L., Wei H.P. (2020). Gemcitabine, lycorine and oxysophoridine inhibit novel coronavirus (SARS-CoV-2) in cell culture. Emerg. Microbes Infect..

[15] Ka S., Merindol N., Sow A.A., Singh A., Landelouci K., Plourde M.B., Pepin G., Masi M., Di Lecce R., Evidente A. (2021). Amaryllidaceae Alkaloid Cherylline Inhibits the Replication of Dengue and Zika Viruses. Antimicrob. Agents Chemother..

[16] Masi M., Di Lecce R., Merindol N., Girard M.P., Berthoux L., Desgagne-Penix I., Calabro V., Evidente A. (2022). Cytotoxicity and Antiviral Properties of Alkaloids Isolated from *Pancratium maritimum*. Toxins.

[17] Girard M.-P., Merindol N., Berthoux L., Desgagné-Penix I. (2022). Le pouvoir antiviral des alcaloïdes de végétaux contre les virus à ARN. Virologie.

[18] Girard M.P., Karimzadegan V., Heneault M., Cloutier F., Berube G., Berthoux L., Merindol N., Desgagne-Penix I. (2022). Chemical Synthesis and Biological Activities of Amaryllidaceae Alkaloid Norbelladine Derivatives and Precursors. Molecules.

[19] Vaněčková N., Hošt‘álková A., Šafratová M., Kuneš J., Hulcová D., Hrabinová M., Doskočil I., Štěpánková Š., Opletal L., Nováková L. (2016). Isolation of Amaryllidaceae alkaloids from Nerine bowdenii W. Watson and their biological activities. RSC Adv..

[20] Al Mamun A., Maříková J., Hulcová D., Janoušek J., Šafratová M., Nováková L., Kučera T., Hrabinová M., Kuneš J., Korábečný J. (2020). Amaryllidaceae Alkaloids of Belladine-Type from *Narcissus pseudonarcissus* cv. Carlton as New Selective Inhibitors of Butyrylcholinesterase. Biomolecules.

[21] Hostalkova A., Marikova J., Opletal L., Korabecny J., Hulcova D., Kunes J., Novakova L., Perez D.I., Jun D., Kucera T. (2019). Isoquinoline Alkaloids from Berberis vulgaris as Potential Lead Compounds for the Treatment of Alzheimer’s Disease. J. Nat. Prod..

[22] Nachon F., Carletti E., Ronco C., Trovaslet M., Nicolet Y., Jean L., Renard P.Y. (2013). Crystal structures of human cholinesterases in complex with huprine W and tacrine: Elements of specificity for anti-Alzheimer’s drugs targeting acetyl- and butyryl-cholinesterase. Biochem. J..

[23] Park J.B. (2014). Synthesis and characterization of norbelladine, a precursor of Amaryllidaceae alkaloid, as an anti-inflammatory/anti-COX compound. Bioorg. Med. Chem. Lett..

[24] Mamun A.A., Pidany F., Hulcova D., Marikova J., Kucera T., Schmidt M., Catapano M.C., Hrabinova M., Jun D., Muckova L. (2021). Amaryllidaceae Alkaloids of Norbelladine-Type as Inspiration for Development of Highly Selective Butyrylcholinesterase Inhibitors: Synthesis, Biological Activity Evaluation, and Docking Studies. Int. J. Mol. Sci..

[25] Darvesh S., Hopkins D.A., Geula C. (2003). Neurobiology of butyrylcholinesterase. Nat. Rev. Neurosci..

[26] Li S., Li A.J., Travers J., Xu T., Sakamuru S., Klumpp-Thomas C., Huang R., Xia M. (2021). Identification of Compounds for Butyrylcholinesterase Inhibition. SLAS Discov. Adv. Sci. Drug Discov..

[27] Kanzari-Mnallah D., Salhi S., Knorr M., Kirchhoff J.-L., Strohmann C., Efrit M.L., Akacha A.B. (2024). Synthesis of isomeric β-cycloalkoxyphosphonated hydrazones containing a dioxaphosphorinane ring: Configurational and conformational investigation and molecular docking analysis. J. Mol. Struct..

[28] Padilla-Bernal G., Vargas R., Martínez A. (2023). Salt bridge: Key interaction between antipsychotics and receptors. Theor. Chem. Acc..

[29] Friedli M.J., Inestrosa N.C. (2021). Huperzine A and Its Neuroprotective Molecular Signaling in Alzheimer’s Disease. Molecules.

[30] Armarego W.L. (2022). Purification of Laboratory Chemicals: Part 2 Inorganic Chemicals, Catalysts, Biochemicals, Physiologically Active Chemicals, Nanomaterials.

[31] Zippilli C., Botta L., Bizzarri B.M., Nencioni L., De Angelis M., Protto V., Giorgi G., Baratto M.C., Pogni R., Saladino R. (2021). Laccase-Catalyzed 1,4-Dioxane-Mediated Synthesis of Belladine N-Oxides with Anti-Influenza A Virus Activity. Int. J. Mol. Sci..

[32] Salentin S., Schreiber S., Haupt V.J., Adasme M.F., Schroeder M. (2015). PLIP: Fully automated protein-ligand interaction profiler. Nucleic Acids Res..

[33] Fischl W., Bartenschlager R. (2013). High-throughput screening using dengue virus reporter genomes. Methods Mol. Biol..

[34] He J., Chen Y., Farzan M., Choe H., Ohagen A., Gartner S., Busciglio J., Yang X., Hofmann W., Newman W. (1997). CCR3 and CCR5 are co-receptors for HIV-1 infection of microglia. Nature.

[35] Chatel-Chaix L., Bartenschlager R. (2014). Dengue virus- and hepatitis C virus-induced replication and assembly compartments: The enemy inside—Caught in the web. J. Virol..

